# Proton Beam Therapy Alone for Intermediate- or High-Risk Prostate Cancer: An Institutional Prospective Cohort Study

**DOI:** 10.3390/cancers10040116

**Published:** 2018-04-10

**Authors:** Takeshi Arimura, Takashi Yoshiura, Kyoko Matsukawa, Naoaki Kondo, Ikumi Kitano, Takashi Ogino

**Affiliations:** 1 Medipolis Proton Therapy and Research Center, 4233 Higashikata, Ibusuki, Kagoshima 8910304, Japan; matsukawa-kyoko@medipolis.org (K.M.); kondo-naoaki@medipolis.org (N.K.); kitano-ikumi@medipolis.org (I.K.); ogino-takashi@medipolis.org (T.O.); 2 Department of Radiology, Kagoshima University Graduate School of Medical and Dental Sciences, 8-35-1 Sakuragaoka, Kagoshima, Kagoshima 8908520, Japan; yoshiura@m3.kufm.kagoshima-u.ac.jp

**Keywords:** prostate cancer, proton beam therapy, efficacy, adverse events, quality of life, sexual function, medical expenses

## Abstract

The role of proton beam therapy (PBT) as monotherapy for localized prostate cancer (PCa) remains unclear. The purpose of this study was to evaluate the efficacy and adverse events of PBT alone for these patients. Between January 2011 and July 2014, 218 patients with intermediate- and high-risk PCa who declined androgen deprivation therapy (ADT) were enrolled to the study and were treated with PBT following one of the following protocols: 74 Gray (GyE) with 37 fractions (fr) (74 GyE/37 fr), 78 GyE/39 fr, and 70 GyE/28 fr. The 5-year progression-free survival rate in the intermediate- and high-risk groups was 97% and 83%, respectively (*p* = 0.002). The rate of grade 2 or higher late gastrointestinal toxicity was 3.9%, and a significant increased incidence was noted in those who received the 78 GyE/39 fr protocol (*p* < 0.05). Grade 2 or higher acute and late genitourinary toxicities were observed in 23.5% and 3.4% of patients, respectively. Our results indicated that PBT monotherapy can be a beneficial treatment for localized PCa. Furthermore, it can preserve the quality of life of these patients. We believe that this study provides crucial hypotheses for further study and for establishing new treatment strategies.

## 1. Introduction

Prostate cancer (PCa) is the second most frequently diagnosed cancer in men worldwide after lung cancer, while it is second after gastric cancer in Japan; PCa accounts for approximately 15% of all malignancies [1,2,3]. PCa-related mortality rates rank fifth worldwide and seventh in Japan. Its mortality rates have generally been declining due to improved treatments and early detection modalities in first-world countries [4], but the incidence and mortality rates of PCa significantly differ among regions.

Since the introduction of prostate specific antigen (PSA)-based screening in the 1980s and 1990s, more patients with PCa were diagnosed at an earlier stage. Furthermore, more than 90% of newly diagnosed PCa cases in the United States in 2004 were localized to the prostate [5]. As such, local treatment modalities for PCa have become increasingly important and resulted in the development of several options including surgery, external beam radiation therapy (EBRT), and brachytherapy [6].

Although androgen deprivation therapy (ADT) has been widely used as the primary treatment modality for the management of localized PCa, particularly among elderly patients [7], ADT does not have cytocidal effects for PCa. Instead, most patients treated with ADT develop refractory disease within a few years; it can also cause adverse effects such as metabolic, cardiovascular, and mental disorders or sexual dysfunction [8,9,10,11,12,13].

EBRT was developed via technological and biological development [14,15] and has become one of the most common treatments for patients with PCa who refuse surgical resection [5]. Many clinical trials have demonstrated the survival benefits of ADT in EBRT with X-ray; thus, EBRT is often used in the management of localized PCa in conjunction with ADT [16,17,18].

Proton beam therapy (PBT) is one type of EBRT. However, proton beams have several features distinct from those of X-rays, such as a sharp dose gradient [19,20]. Several recent studies showed favorable results of PBT for localized PCa; however, these studies included patients who also underwent ADT, and the role of PBT in these patients remains unclear.

The purpose of this study was to evaluate the efficacy and adverse events of PBT monotherapy for intermediate or high-risk PCa and to explore the possibility of cure without ADT, possibly eliminating superfluous medical expenses and adverse effects including sexual dysfunction.

## 2. Results

### 2.1. Patient Characteristics

The patient characteristics are summarized in Table 1. The patients’ median age was 65 (range, 39–86 years). All patients had good performance status (Eastern Cooperative Oncology Group (ECOG) score ≤ 1) and were followed up for at least 2 years, with a median follow-up time of 52 months. The intermediate- and high-risk groups comprised 112 (55%) and 92 (45%) patients, respectively. The 70 GyE/28 fr, 74 GyE/37 fr, and 78 GyE/39 fr protocols were performed in 61 (30%), 85 (42%), and 58 (28%) patients, respectively. Thirty patients (15%) were administered with anticoagulants during the treatment due to comorbidities, and the levels of glycated hemoglobin (HbA1c) before PBT were elevated in 27 patients (13%). The median percentage of positive biopsy cores was 25 (range, 5–100%) and perineural invasion was observed in biopsy specimens from eleven (5%) patients. The median clinical target volume (CTV)/planning target volume (PTV) D95 (dose that covers 95% of CTV/PTV) and CTV/PTV V95 (percent volume that received at least 95% of the prescribed dose) were 74 (range, 69–78)/59 (range, 38–67) GyE and 100 (range, 98–100)/79 (range, 63–88) %, respectively. The median time to reach PSA nadir in the 70 GyE/28 fr, 74 GyE/37 fr, and 78 GyE/39 fr protocols was respectively 40 (range, 4–53) months, 46 (range, 1–75) months, and 49 (range, 4–74) months, and their corresponding values were 0.5 ng/mL (range, 0.0–2.8), 0.5 ng/mL (range, 0.1–2.4) and 0.3 ng/mL (range, 0.0–3.2).

### 2.2. Efficacy

**Figure 1:**
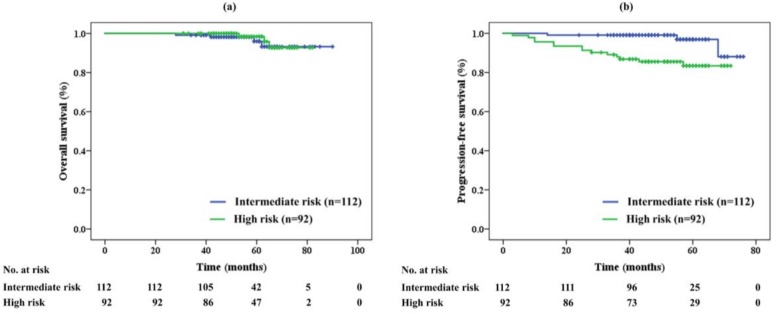
(**a**) Overall survival and (**b**) progression-free survival of proton beam therapy alone in each risk group

The overall survival (OS) rate in each risk group is shown in Figure 1a. The 5-year OS was 96% and 98% in the intermediate- and high-risk groups, respectively. No significant difference was noted between the two groups (*p* = 0.673). Seven patients (3%) died during the study period; one patient in the high-risk group died of PCa 53 months after PBT, while the other six patients died from another disease unrelated to PCa.

Figure 1b shows the progression-free survival (PFS) rates. The 5-year PFS was significantly different between the two groups (*p* = 0.002) at 97% in the intermediate-risk and 83% in high-risk group. Seventeen patients (8%) relapsed after PBT. Three (3%) and eight (9%) patients in the intermediate- and high-risk groups, respectively, were diagnosed with biochemical recurrence. Meanwhile, six patients in the high-risk group were diagnosed with metastasis in the lymph node (*n* = 2, 2%) and bone (*n* = 2, 2%), and by clinical decision by self-referring urologists (*n* = 2, 2%).

Table 2 shows univariate and multivariate analyses for PFS. Although multivariate analysis identified PSA (<12 vs. 12≤, *p* = 0.003), Gleason score (≤7 vs. 8≤, *p* = 0.001) and clinical T stage (≤2 vs. 3, *p* = 0.019) as significant prognostic factors, there were no significant differences in PFS based on patient age, percentage of positive biopsy cores, existence of perineural invasion in biopsy specimens, total dose, dose per fraction, PTV D95, and PTV V95.

### 2.3. Adverse Events

**Figure 2:**
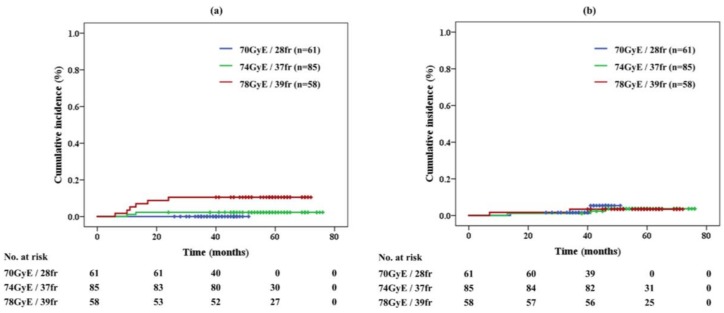
(**a**) Late gastrointestinal toxicity and (**b**) genitourinary toxicity of proton beam therapy alone in each protocol

No grade 2 or higher GI toxicity was seen in the acute phase (Table 1). In total, 3.9% of patients developed grade 2 late gastrointestinal (GI) toxicity, which developed within 6 months to 2 years after the treatment. The cumulative incidence of late GI toxicity was 10.5% in 78 GyE/39 fr, 2.3% in 74 GyE/37 fr, and 0.0% in 70 GyE/28 fr, respectively (Figure 2a). Significant differences in late GI toxicities were noted between the protocols of 70 GyE and 78 GyE (*p* = 0.010) and between 74 GyE and 78 GyE (*p* = 0.037). Grade 3 or higher GI toxicity was not observed in the study. All patients with grade 2 late GI toxicity presented with rectal hemorrhage and received cauterization and/or hyperbaric-oxygen therapy.

Eleven (19%) out of the 57 patients who received a total dose of 78 GyE irradiation were administered anticoagulant agents during PBT, and one (9%) patient developed GI toxicity. We did not observe specific impact of anticoagulants on late GI toxicity in PBT (*p* = 0.872).

Six patients (11%) in the 78 GyE group had elevated HbA1c before PBT, but a significant increase in the rate of late GI toxicity was not observed in these patients (*p* = 0.097).

The rates of acute and late genitourinary (GU) toxicities were 23.5% and 3.4%, respectively. The late GI toxicities developed with 7 to 46 months after the treatment. The cumulative incidence of acute and late GU toxicities was respectively 28.1% and 3.4% in the 78 GyE protocol, 27.9% and 3.5% in 74 GyE, and 13.1% and 3.3% in 70 GyE. The incidence of acute GU toxicity was significantly different between the 70 GyE and 74 GyE protocols (*p* = 0.033) and between the 70 GyE and 78 GyE protocols (*p* = 0.045). The cumulative incidence of grade ≥2 late GU toxicity for each protocol is shown in Figure 2b.

The details of grade 2 GU toxicity are shown in Table 1. The incidences of grade 2 GU toxicity in the acute and late phases were respectively 30 (15%) and 3 (1%) in retention, 21 (10%) and 1 (<1%) in frequency, 10 (5%) and 0 (0%) in pain, 4 (2%) and 0 (0%) in urgency, 0 (0%) and 4 (2%) in hematuria and 0 (0%) and 1 (<1%) in incontinence. Patients with grade 2 GU toxicity were treated with α1-adrenergic receptor antagonists and/or non-steroidal anti-inflammatory agents for urinary disturbances, and with cauterization and/or hyperbaric-oxygen therapy for hematuria.

### 2.4. Sexual QOL

**Figure 3:**
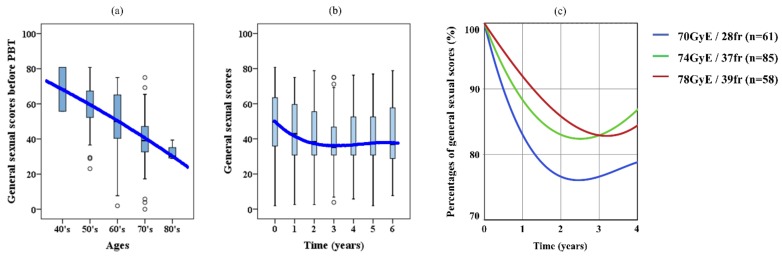
(**a**) Aging variation of general sexual scores in the expanded prostate cancer index composite. (**b**) Chronological variation of scores after proton beam therapy. (**c**) Approximate curves expressed as percentages after proton beam therapy in each protocol.

The general sexual scores in expanded prostate cancer index composite (EPIC), grouped according to age, are shown in Figure 3a. The bold blue line represents the approximate curve of average aging variation, and the approximate formula was expressed with the quadratic approximation

S= −0.003y^2^ − 0.531y + 96.360 (R^2^ = 0.997, S: score; y: years). (1)

The general sexual scores gradually decreased with age, and the average score for patients in their 40’s was around 70 and declined to approximately 30 for patients in their 80’s.

The chronological variation of EPIC scores after PBT is shown in Figure 3b. The approximate formula was represented with the cubic approximation

S = −0.143y^3^ + 1.876y^2^ − 7.529y +49.152 (R^2^ = 0.991). (2)

The score was lowest 3 years after the treatment, and then it recovered slightly.

Figure 3c shows the approximate curves expressed as percentages after PBT in each protocol

70 GyE: S = −0.007y^3^ + 0.074y^2^ − 0.237y (R^2^ = 0.999); (3)

74 GyE: S = −0.002y^3^ + 0.038y^2^ − 0.153y (R^2^ = 0.999); (4)

78 GyE: S = 0.002y^3^ +0.004y^2^ − 0.087y (R^2^ = 0.882). (5)

The local minimum points for the 70 GyE, 74 GyE, and 78 GyE protocols were respectively −24.0%, −17.6%, and −17.2%, at 2.5 years, 2.5 years, and 3.2 years after the treatment.

### 2.5. Costs of PBT

Table 3 shows the estimated total costs of PBT (until March 2018 and since April 2018), EBRT plus ADT over 2.5 years, and brachytherapy (BT) plus EBRT plus ADT over 2 years in Japan. Additional expenses such as treatment measures against adverse events and follow-up after the treatment were not included in the calculation.

The price of PBT varied from facility to facility in Japan until March 2018. The costs of EBRT with intensity-modulated radiation techniques and with 3-dimensional irradiation procedures were estimated as a conventional radiotherapy with 35 to 39 fractions and with 25 fractions, respectively. For BT, the cost was estimated for the implantation of 60 to 100 iodine-125 seeds. The costs of ADT were calculated with a combination of luteinizing hormone-releasing hormone agonists and anti-androgens, and its range was based on the difference between patented and generic drugs.

## 3. Discussion

PCa is generally slow-growing and might not be a life-threatening disease, particularly for elderly patients at an early stage [21]. Although numerous therapeutic approaches have been implemented, and several characteristic treatments are currently used in clinical practice [6], the optimal patterns of treatment for specific statuses of the disease are unknown because even long-term and large-scale randomized prospective studies have not shown significant differences in the common treatment modalities [22], and each clinical trial has required a 10-years or longer follow-up period to achieve the final results.

Previously, EBRT could not safely deliver doses greater than 70 Gy to the prostate because of limitations in reducing the irradiation to adjacent organs, which resulted in local control rates of less than 65% [23]. Thus, EBRT was started to be used in conjunction with ADT, which is easily administered even though the definitive roles of ADT with X-ray in the treatment of PCa remain unknown [24,25]. Treatment outcomes have been improved with advances in radiation technology over the past two decades, and many clinical studies have demonstrated the benefits of EBRT combined with ADT [16,18,26]. However, in the recent high-dose radiotherapy era, some trials did not observe any superiority of ADT for localized PCa [25,27].

Yang et al. [25] identified 46,325 patients in the National Cancer Database with high-risk PCa who were treated with EBRT, with or without BT and ADT, and reported that ADT might not improve survival for those patients who undergo the combined treatments. Locally elevated doses with EBRT and BT but without ADT appeared to have improved local control for PCa and have led to favorable results [28].

X-rays and γ-rays penetrate through the prostate and irradiate surrounding organs such as the rectum and bladder. On the other hand, proton beams possess a characteristic Bragg peak [19], which allows localization of the irradiation dose to the prostate, thus minimizing damage to adjacent tissues [29]. Therefore, we consider PBT to be among the most promising therapeutic procedures for PCa, and we believe that PBT might lead to favorable results in the treatment of localized PCa even without ADT.

The 5-year OS rates in the present study were approximately 96% and 98% in the intermediate- and high-risk groups, respectively. Meanwhile, the OS in previous reports on PBT for PCa was between 88% and 97% for the intermediate-risk group and between 86% and 96% for the high-risk group [30,31,32,33], which was similar to our outcomes (Table 4). Although one patient (0.5%) whose Gleason score was 5 + 5 died of PCa 53 months after the treatment, we recognized that these results were ethically acceptable and demonstrated the value of PCa monotherapy as an adequate and effective treatment modality for patients with intermediate- and high-risk PCa.

Our study revealed that the 5-year PFS for patients with localized PCa who underwent PBT alone was 97% and 83% in the intermediate- and high-risk groups, respectively. Table 4 shows the previous results of EBRT for localized PCa. The PFS in the intermediate- and high-risk groups was 83–86% and 68–72% for intensity-modulated radiation therapy (IMRT), 91–99% and 74–86% for PBT, and 97% and 88% for carbon ion beam therapy. Our results were not inferior to those of previous studies that included ADT.

Compared with the two reports with a radiation protocol from Memorial Sloan-Kettering Cancer Center [34,35], there was almost no difference in PFS between both risk groups despite the differences in the follow-up period and number of patients. Therefore, although the follow-up period was shorter, and the number of patients was smaller, our results were comparable to those of previous reports.

The rate of grade 2 late GI toxicity was 3.9%, and all these occurred within 2 years after PBT. All patients in the study were followed up for at least 2 years, and we believe that this result was close to the final figure for the late GI toxicity of PBT. Although our total results were similar to those of previous reports, substantial variations between the protocols were observed. A total of 10.5% of patients who were irradiated with a total dose of 78 GyE had a grade 2 GI toxicity; on the other hand, no patient irradiated with 70 GyE developed these toxicities, suggesting that the total irradiation dose might have a stronger effect on the occurrence of GI toxicity than the dose per fraction.

The rate of late GU toxicity was 3.4%, and it developed within 7 months to 46 months after PBT. The rate of late GU toxicity was not significantly different between the three PBT protocols. These data suggested that the rate of GU toxicity might have been underestimated, and a longer follow-up period is needed to determine the final figure of GU toxicity in PBT. However, our results seemed to be superior to those of previous reports on IMRT.

The incidence of GU toxicity in the acute phase was 28.1% for the 78 GyE/39 fr protocol, 27.9% for 74 GyE/37 fr, and 13.1% for 70 GyE/28 fr. The rate of acute GU toxicity was significantly lower for the 2.5 GyE per fraction protocol than for the 2 GyE per fraction protocol, indicating that in PBT, the total dose might have more impact on acute GU toxicity than the dose per fraction. Compared with the results reported by Cahlon et al., no sufficient advantages of IMRT to PBT in terms of acute GU toxicity were noted.

In evaluating the sexual scores with EPIC, we first examined the aging variation so we could understand the sexual status according to age. The sexual function scores decreased with age, and the average score in the 40’s age group was less than half that in the 80’s. Accordingly, when discussing sexual function, we might have to consider that the changes in scores over the years after PBT have been influenced by not only PBT, but also aging.

Regarding the chronological variation of EPIC scores after PBT, the lowest point was about 3 years after the treatment, and then the score recovered slightly. Although a similar trend was observed for every protocol, slight differences in each were also noted. The scores for the 70 GyE protocol showed the fastest and largest decrease, and the range of reduction was almost the same for the 2 GyE per fraction protocol. The time to the lowest point was the longest for the total dose of 78 GyE. These results indicated that the dose per fraction might have more influence on the absolute decrease in the score, while the total dose has a greater impact on the time to the lowest point.

Our results were similar to those of Resnick et al. who presented their findings in graphs [38]. This method might allow for the prediction of the approximate change of sexual function after EBRT. For example, in men with an average age of 65 who underwent 70 GyE/28 fr PBT, when calculated using the Equations (1) and (3), the EPIC score of sexual function would be lowered by 21.2% at 4 years after the treatment, where 7.6% is accounted for by aging and 13.6% is from damage from PBT.

Table 3 shows that the estimated medical expenses related to PBT were almost the same as those of the two treatment options for localized PCa in Japan, i.e., EBRT plus ADT over 2.5 years and BT plus EBRT plus ADT over 2 years. Because up to March 2018 the Japanese government had not provided financial support for PBT, unlike other treatments, private insurance companies had supported these expenditures instead. However, the government has announced that national financial support would be provided for PBT as of April 2018, although an extremely low price was set. Although PBT is often deemed to be expensive worldwide [39,40], we believe that the cost issue might be resolved if we did not perform mandatory ADT with PBT for localized PCa.

It should be noted that this study was conducted at an institution in Japan and any application of our findings should be adapted to the specific situation of each country. Furthermore, as this study was exploratory and had no control group, our results are suggestive but could not be confirmed. However, we believe that this type of study is extremely important for generating hypotheses that lead to further study, and for establishing new treatment strategies [41,42].

## 4. Materials and Methods

### 4.1. Patients and Study Design

Patients who met the following criteria were eligible for the study: (1) pathologically confirmed prostate biopsy; (2) no metastasis with computed tomography (CT) and bone scintigraphy, and/or positron-emission tomography (PET) and/or magnetic resonance imaging (MRI) within 3 months before treatment; (3) no prior malignancy within 5 years; (4) no history of surgery and radiotherapy in pelvis; (5) life expectancy of greater than 5 years with ECOG performance status of ≤1 (6) rectal endoscopy within 3 months and endoscopic mucosal resection (EMR) for polyps that were detected on the abdominal wall of the rectum at least 2 weeks before treatment.

Primary endpoints of the study were the efficacy and toxicity of PBT monotherapy. Secondary endpoints included comparisons of the treatment protocols, sexual QOL and medical costs.

Between January 2011 and July 2014, 350 consecutive patients with localized PCa who were stratified into intermediate- or high-risk groups according to Union for International Cancer Control (UICC) 7th edition and the D’Amico risk classification were admitted to our center [43]. Of these, 128 chose PBT in combination with ADT according to the National Comprehensive Cancer Network (NCCN) guidelines [6], while the remaining 218 patients declined ADT and were enrolled in the study.

**Figure 4:**
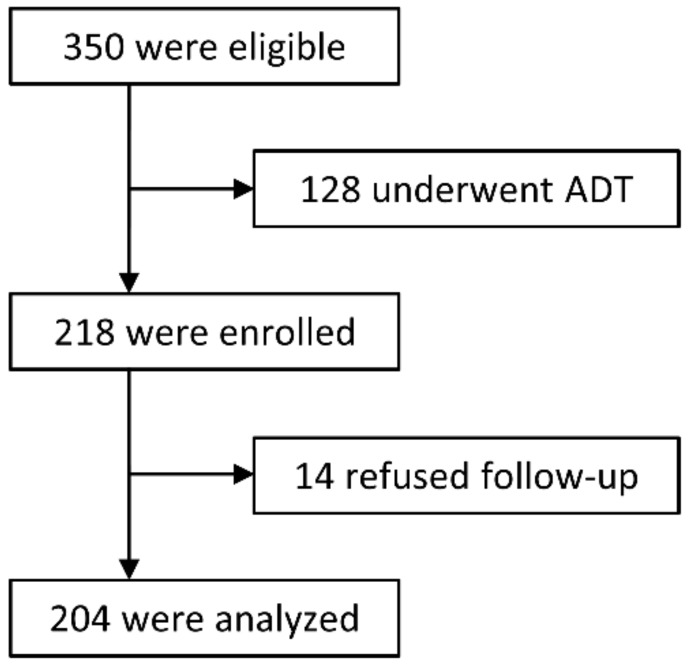
Trial flowchart.

This study was approved by the institutional review board (MEDI 10-2 and 13-5), and written informed consent was obtained from all patients prior to enrollment. All procedures were performed in accordance with the ethical standards outlined in the Helsinki Declaration of 1975, as revised in 2000. A flowchart of the study is shown in Figure 4.

Patients were all required to receive PSA testing within 3 months after PBT. ADT had to be initiated immediately when patients developed biochemical failure or clinical recurrence during the follow-up. Moreover, we were required to monitor the outcomes of this study continuously and discontinue the trial when our results appeared to be worse than those of previous reports on EBRT with X-ray. We determined the definitive borderline for biochemical relapse-free survival as 80% and 65% in the intermediate- and high-risk groups, respectively.

### 4.2. PBT

All patients were immobilized with a thermoplastic cast in the supine position and underwent computed tomography (CT) (with 2-mm-slice thickness) and magnetic resonance imaging (MRI) (3-mm-slice thickness). The treatment plan was developed with a CT-based 3-dimensional treatment planning system (XiO–M, ELEKTA, Stockholm, Sweden) using a fusion imaging technique.

The gross tumor volume was not indicated for all patients, but the CTV was defined to the prostate gland for intermediate-risk patients, and the base of the seminal vesicle was added to the CTV for high-risk patients. However, a whole volume was defined for higher-risk patients (T3b or PSA ≥ 50). The PTV was set as the CTV plus a 10-mm margin in all directions, but 7-mm bilaterally.

We applied the relative biological effect value for PBT of 1.1 and described the doses of PBT as GyE [44]. Although we performed PBT using 74 GyE for intermediate-risk patients and 78 GyE for high-risk patients with a conventional dose of 2 GyE per fraction at the beginning (74 GyE/37 fr and 78 GyE/39 fr, respectively), a protocol of 70 GyE with 28 fractions (70 GyE/28 fr) was initiated for patients in both risk groups in 2013 by considering the rationale of lower α/β ratio for PCa [14,15].

The dose constraints for the rectum were V50 < 55%, V60 < 40%, and V70 < 25% [45]. The maximal doses of the colon and small intestine were 61 GyE and 55 GyE, respectively [36]. We did not define a specific constraint for the bladder. These values were applied for the 74 GyE and 78 GyE protocols and were modified for the 70 GyE irradiation protocol in accordance with the Linear-Quadratic model [46].

All patients were treated with 210 MeV horizontal proton beams, which were produced by a beam-wobbling system for a flatter irradiated field and with a ridge filter to form a spread-out Bragg peak (Mitsubishi Electric Corporation, Tokyo, Japan). The patient set-up was performed daily by subtracting the two sets of orthogonal digital radiographs before irradiation. Set-up errors were compensated for by the positioning system and movements of the treatment couch within 1 mm from the position in digitally reconstructed radiographs.

All patients were administered with magnesium oxide to prevent constipation and with dimethicone for reducing intestinal gas during PBT. Moreover, they were required to defecate 30 min before proton beam irradiation, and also when gas in the rectum was observed immediately before irradiation. However, we did not use image-guided techniques with fiducial markers, spacers, or rectal balloons to decrease the irradiated volume of the rectum.

### 4.3. Follow-Up and Evaluation of Adverse Effects

All patients were evaluated for PSA, adverse events, and quality of life (QOL) every 3 months for the first 3 years after PBT. The follow-up period was then extended for 6 months maximum by self-referring urologists. We calculated the number of days and terms from the last day of PBT for all events and occurrences.

Biochemical failure was determined based on the Phoenix definition, and clinical recurrence was decided via imaging modalities such as bone scintigraphy, CT, positron emission tomography, and MRI [47]. Meanwhile, ADT was initiated if recurrence developed as defined by a self-referring urologist even if the value of PSA did not meet the necessary increase.

Adverse events were evaluated in accordance with the National Cancer Institute Common Terminology Criteria of Adverse Events 4th edition, and the EPIC was used to evaluate QOL in sexual function [48].

### 4.4. Costs

We estimated the total costs of PBT and compared them to those of the other treatments specified in the NCCN clinical guideline for patients with PCa [6]. Since medical fees were strictly regulated by the Japanese Ministry of Health, Labour and Welfare, we calculated the medical expenditures based on the national fee schedule.

### 4.5. Statistical Analysis

Statistical analyses were performed for patients with PCa who were followed up for at least 2 years after PBT. We estimated the rates of OS, PFS, and GI/GU toxicities using the Kaplan-Meier method, two-sided log-rank test, and the Mann-Whitney U test with IBM SPSS Statistics software version 24.0 (IBM, Inc., Armonk, NY, USA). To assess the effects of prognostic factors, Kaplan-Meier analyses for PFS were performed based on patient age, PSA, Gleason score, percentage of positive biopsy cores, perineural invasion in biopsy specimens, clinical T stage, dose/fraction, D95 and V95 to CTV/PTV using log-rank tests. Cox proportional hazards analysis was performed for multivariate analyses of selected factors. Statistical analyses were performed with SAS Release 9.4 (SAS Inst., Cary, NC, USA).

We also evaluated the scores of general sexual function with EPIC before and after PBT with multiple regression analyses. The scores measured via EPIC were calculated according to the instrument instructions [48]. The scores ranged from 0 to 100, with high scores representing more favorable QOL. A *p* value of <0.05 was considered statistically significant. 

## 5. Conclusions

We performed a prospective cohort study of 218 patients with intermediate- and high-risk PCa who underwent PBT and continued to decline ADT until disease recurrence. Moreover, we evaluated the efficacy and adverse events of PBT. Our results were similar to those of previous studies on PBT combined with ADT. We found that PBT monotherapy can be a beneficial treatment modality for these patients and may maintain the QOL of these men. However, studies that include more patients and longer follow-up are needed to clarify the definitive role of PBT in the treatment of localized PCa.

## Figures and Tables

**Table 1:** Patient characteristics.

Factors		Items	Value	Unit
Age		Median	65	years
		Range	39–86	years
Performance status (ECOG)		0	200	(98%)
		1	4	(2%)
Follow-up time		Median	52	months
		Range	24–76	months
Initial PSA (ng/ml)		<10	140	(69%)
		10–20	44	(22%)
		≥20	20	(10%)
Gleason score		6	18	(9%)
		7	125	(61%)
		8	41	(20%)
		≥9	20	(10%)
Clinical T stage (UICC)		T1c	88	(43%)
		T2a	70	(34%)
		T2b	11	(5%)
		T2c	18	(9%)
		T3a	14	(7%)
		T3b	3	(1%)
Risk stratification		Intermediate	112	(55%)
		High	92	(45%)
Prescription dose (GyE)		70	26	(Intermediate, 13%)
			35	(High, 17%)
		74	85	(42%)
		78	58	(28%)
Anticoagulants		Yes	30	(15%)
		No	174	(85%)
HbA1c		<6.5	177	(87%)
		≥6.5	27	(13%)
Percentage of positive biopsy cores		Median	25	%
		Range	5–100	%
Perineural invasion in biopsy specimens		Yes	11	(5%)
		No	193	(95%)
CTV D95		Median	74	GyE
		Range	69–78	GyE
PTV D95		Median	59	GyE
		Range	38–67	GyE
CTV V95		Median	100	%
		Range	98–100	%
PTV V95		Median	79	%
		Range	63–88	%
GU toxicity (grade 2)	Late	Median	34	months
		Range	7–46	months
	Acute/Late	Retention	30/3	(15%/1%)
		Frequency	21/1	(10%/<1%)
		Pain	10/0	(5%/0%)
	Events	Urgency	4/0	(2%/0%)
		Hematuria	0/4	(0%/2%)
		Incontinence	0/1	(0%/<1%)
GI toxicity (grade 2)	Late	Median	12	months
		Range	6–24	months
Events	Acute/Late	Hemorrhage	0/8	(0%/4%)
PSA nadir (Value)	70(GyE)	Median	0.5	ng/ml

**Table 2:** Univariate and multivariate analysis for PFS.

Factors	Univariate		Multivariate	
	5-Year PFS	*p* Value	Hazard Ratio (95% CI)	*p* Value
**Age**				
<60 vs. ≥60	92% vs. 91%	0.989	–	–
<70 vs. ≥70	89% vs. 94%	0.843	1.595 (0.482–5.282)	0.445
**PSA**				
<10 vs. ≥10	93% vs. 86%	0.052	–	–
<12 vs. ≥12	93% vs. 82%	0.019	0.185 (0.061–0.555)	0.003
<20 vs. ≥20	92% vs. 79%	0.055	–	–
**Gleason score**				
≤6 vs. ≥7	100% vs. 90%	0.535	–	–
≤7 vs. ≥8	94% vs. 84%	0.005	0.122 (0.034–0.441)	0.001
**Percentage of positive biopsy cores (%)**				
<20 vs. ≥20	94% vs. 89%	0.342	–	–
<30 vs. ≥30	94% vs. 87%	0.146	0.546 (0.199–1.501)	0.241
**Perineural invasion in biopsy specimens**				
Yes vs. No	91% vs. 91%	0.955	1.992 (0.209–18.995)	0.549
**Clinical T stage (UICC)**				
1 vs. ≥2	98% vs. 86%	0.011	–	–
≤2 vs. 3	92% vs. 76%	0.015	0.221 (0.063–0.778)	0.019
**Dose (GyE/fraction)**				
70/28 vs. 74/37	93% vs. 92%	0.281	1.822 (0.362–9.172)	0.467
74/37 vs. 78/39	92% vs. 88%	0.296	3.231 (0.778–13.413)	0.106
70/28 vs. 78/39	93% vs. 88%	0.325	1.773 (0.460–6.841)	0.406
70/28 vs. 74/37 & 78/39	93% vs. 91%	0.966	–	–
**PTV D95 (GyE)**				
<60 vs. ≥60	89% vs. 91%	0.634	1.197 (0.350–4.089)	0.775
**PTV V95 (%)**				
<80 vs. ≥80	90% vs. 92%	0.834	0.433 (0.137–1.363)	0.152

Abbreviations: PFS, progression-free survival; CI, confidence interval; PSA, prostate specific antigen; UICC, Union for International Cancer Control; GyE, Gray equivalent; PTV, planning target volume; D95, dose that covers 95% of the volume; V95, percent volume that received at least 95% of the prescription dose.

**Table 3:** Comparison of estimated total costs in 3 patterns of non-operative treatments for localized prostate cancer

Currency	Range	PBT (Until March 2018)	EBRT (IMRT) + ADT 2.5y	BT + EBRT (3D CRT) + ADT 2y	PBT (Since April 2018)
Japanese yen	Minimum	¥2,600,000-	¥2,522,100-	¥2,591,380-	¥1,600,000-
	Maximum	¥3,000,000-	¥3,123,280-	¥3,158,040-	
US dollar	Minimum	$21,667-	$21,018-	$21,595-	$13,333-
	Maximum	$25,000-	$26,027-	$26,317-	

Abbreviations: PBT, Proton beam therapy; EBRT, External beam radiotherapy; IMRT, Intensity modulated radiation therapy; ADT, Androgen deprivation therapy; y, years; BT, Brachytherapy; 3D CRT, 3-dimensional radiation therapy; US dollar = 120 Japanese yen.

**Table 4:** Comparison with previous reports on radiotherapy for intermediate- and high-risk PCa.

Reports	Year	EBRT	Patients (Number)	Median f/u (Months)	Dose (Gy(E))	Fraction	ADT (%)	5-Year Recurrence-Free Survival (%)		Gr 2 (Gr 3) Toxicity (%)		
								Intermediate Risk	High Risk	Acute GU	Late GU	Late GI
Kupelian et al. [36]	2007	IMRT	508	45	70	28	60 *	83	72	–	7.0	6.0
Cahlon et al. [34]	2008	IMRT	378	53	86.4	48	66 *	85	70	22.0 (0.6)	12.6 (2.5)	3.4 (0.4)
Ishikawa et al. [37]	2012	Carbon	768	43	57.6–66	16–20	100	97	88	–	6.2 (0.1)	1.9 (0.0)
Spratt et al. [35]	2013	IMRT	806	66	86.4	48	66	86 **	68 **	–	18.9 (2.2)	3.7 (0.7)
Mendenhall et al. [30]	2014	Proton	122	62	78–82	39–41	37	99	76	(0.0)	(0.9)	(0.5)
Bryant et al. [31]	2016	Proton	780	66	72–80	36–43	27	94	74	(1.8)	(2.9)	(0.6)
Takagi et al. [32]	2017	Proton	1126	70	74–78	37–39	64	91	86	–	1.9 (0.1)	3.8 (0.1)
Iwata et al. [33]	2018	Proton	1076	69	63–80	21–40	68	91	83	–	(0.3)	(0.5)
Our study	2018	Proton	204	52	70–78	28–39	0	97	83	23.5 (0.0)	3.4 (0.0)	3.9 (0.0)

Abbreviations: PCa, prostate cancer; EBRT, external beam radiation therapy; f/u, follow-up; IMRT, Intensity-modulated radiation therapy; Gr, grade; GU, genitourinary; GI, gastrointestinal; * the rate of ADT for patients including low-risk; ** 7-year biochemical relapse-free survival.
